# Design and implementation of a BSN-based system for plantar health evaluation with exercise load quantification

**DOI:** 10.1186/s12938-017-0389-9

**Published:** 2017-08-03

**Authors:** Yang Wang, Zhiwen Liu, Jian Yang, Shaodong Ma

**Affiliations:** 1 School of Information and Electronics, Beijing Institute of Technology, Beijing, China; 2 School of Optoelectronics, Beijing Institute of Technology, Beijing, China

**Keywords:** Plantar pressure, Exercise load quantification, Heart rate, Strike frequency

## Abstract

**Background:**

Plantar pressure measurement has become increasingly useful in the evaluation of plantar health conditions thanks to the recent progression in sensing technology. Due to the large volume and high energy consumption of monitoring devices, traditional systems for plantar pressure measurement are only focused on static or short-term dynamic monitoring. It makes them inappropriate for early detections of plantar symptoms usually presented in long-term activities.

**Methods:**

A prototype of monitoring system based on body sensor network (BSN) is proposed for quantitative assessment of plantar conditions. To further assess the severity of plantar symptoms which can be reflected from the pressure distribution in motion status, an approach to conjoint analysis of pressure distribution and exercise load quantification based on the strike frequency (SF) and heart rate (HR) is also proposed.

**Results:**

An examination was tested on 30 subjects to verify the capabilities of the proposed system. The estimated correlation rate with reference devices ($r>0.9$) and error rate on the average ($R_{AE}<0.08$) of HR and SF indicated equal measuring capabilities as the existing commercial products . Comprised of the conjoint analysis based on HR and SF, the proposed method of exercise load quantification was examined on all subjects’ recordings.

**Conclusions:**

A prototype of an innovative BSN-based bio-physiological measurement system has been implemented for the long-term monitoring and early evaluation of plantar condition. The experimental results indicated that the proposed system has a great potential value in the applications of long-term plantar health monitoring and evaluation.

## Background

Plantar pressure measurement is a common and effective assessment widely applied to plantar health evaluation [1]. Recent studies mostly focused on exploring the relationship between the plantar condition and pressure with static measurement or short-term dynamic monitoring in the clinical environment due to the limitations of measuring devices [2, 3]. However, most slight symptoms of plantar ulceration and bruise usually cannot be detected from short-term and lightweight activities, making the measurement of plantar pressure difficult to be utilized in the early diagnose [4]. Along with the enhancement of the load and duration, potential broken areas can hardly bear more pressure which can be reflected from the change of pressure distribution in the long-term traction [5]. Intensive changes with lightweight exercise usually reveal worse conditions, which is beneficial to the analysis of plantar health. Therefore, it is necessary to quantify the load of exercise for the assessment of symptom severity. The conjoint analysis of plantar pressure distribution and exercise load quantification will play an important role in the evaluation on plantar health conditions.

Traditional equipment is not suitable for long-term dynamic monitoring due to multiple restrictions such as large volume, wire communication and high power consumption [6]. Thanks to the recent progression in sensing technique and wireless communication, wearable solutions based on body sensor network (BSN) have been widely used in healthcare applications. BSN is a kind of wireless sensor networks with physiological sensors for vital monitoring [7]. The embracement of wireless sensing and wearable design may release the use of vital monitoring equipment from indoor environment, which technologically enables long-term healthcare for a huge number of people if the cost of a measuring device is affordable. Gerlach et al. developed a sort of printed pressure sensors for ulcer prevention [8]. Although the designed sensors can relieve the plantar discomfort during exercise, the complex connections between sensors and devices make it inconvenient to walk with. Shu et al. implemented an in-shoe measuring device based on fabric sensors [9]. The simplification of connections provided the possibility for BSN-based monitoring in outdoor environment. However, the lack of exercise load quantification still made it inappropriate for early diagnose of plantar conditions. To our best knowledge, the existing wearable systems for plantar pressure measurement haven’t taken the exercise load quantification into account. Hence, there is an urgent need to develop an integrated system appropriate for conjoint analysis of pressure distribution and exercise load during long-term monitoring.

As the most common activity monitor, pedometers with tri-axial accelerometers have been widely deployed in healthcare devices. Using accelerometers to convert acceleration signals to the strike frequency (SF) and distance by specific algorithms, Fitbit one yielded a high performance on activity monitoring [10]. With the provided applications, exercise information was presented to users and exercise load can be quantified in terms of motion distance. In addition, global positioning system (GPS) is another significant solution in activity monitoring. Worringham et al. developed a GPS-based system that enabled long-term motion speed estimation which can be regarded as an effective approach to exercise load estimation [11]. Although motion features like speed and distance are the most obvious indicators to reflect the intensities, people usually have different tolerance to the same exercise due to different body conditions [12]. It means that exercise load should not be estimated only based on the motion features. Other efficient indicators related to exercise endurance need to be introduced into the load quantification as a supplementary evaluation.

The Borg’s rating of perceived exertion (RPE) has been widely used in the load estimation of activities [13]. This quantified method is based on the self-report questionnaire from subjects, which may bring uncertainties into the quantification due to subjective sensations. As we know, fluctuations of cardiovascular conditions are usually related to the change of exercise intensities [14]. The variabilities of blood pressure (BP) and heart rate (HR) adopted to represent the cardiovascular functions can be utilized to reflect the endurance of activity indirectly [15]. Current approaches to BP and HR estimation are mainly focused on noninvasive measurement, making it possible for long-term monitoring during exercise.

Cuff-based BP monitors are the most common devices adopted in healthcare systems due to its relatively stable property [16], but the procedure of inflation and deflation may disturb users intensively. Cuff-less BP monitoring based on pulse transit time (PTT) is an alternative solution to enhance the convenience and comfort level [17]. However, the interference of motion artifact (MA) has serious impact on the PTT estimation, leading to unreliable BP measurement [18].

Compared with BP, HR is much more appropriate for long-term and continuous monitoring. Lots of HR monitoring systems are implemented based on the analysis of electrocardiography (ECG) because of the relatively better quality of signals [19]. Doherty et al. designed a multi-sensor system for monitoring HR and activities during daily life [20]. Results of the experiments on 40 subjects indicated the effectiveness on HR monitoring based on ECG. Although patch electrode is the most common sensor for ECG acquisition in the clinical or home environment, long-term usage of conductive adhesive may lead to skin allergies. In order to overcome this drawback, textile electrodes and chest bands are used to enhance the biological compatibilities. For the purpose of increasing the comfort level, Cho et al. realized novel textile electrodes for HR monitors [21]. Costa et al. integrated ECG and respiration sensors into a chest band to implement an e-health wearable system [22]. Nevertheless, the main disadvantage of ECG-based monitors is that the locations of sensors must be fixed, bringing extreme inconvenience to the usage of monitoring.

HR monitors based on reflectance-mode photoplethysmography (PPG) have received tremendous attentions in the field of healthcare research due to the advantages of unconstrained sensor locations and various acquisition methods [23]. Jonathan et al. developed a system based on smart phone that can extract HR from PPG via the integrated camera and light-emitting diode (LED) [24]. Some commercial devices like Fitbit also adopt reflectance-mode PPG for HR monitoring [25]. However, MA interference is still an open issue in HR estimation based on PPG. Yousefi et al. proposed a motion-tolerant algorithm proved to be effective for HR estimation on treadmill. Improving the sensor architecture is another way to achieve accurate results [26]. Shimazaki et al. developed a canceller equipped with two reflectance-mode LED/photo diode (PD) sensors to acquire PPG signals and MA information, respectively [27]. The experimental results revealed better performance on MA resistance.

Due to the motivations of pressure measurement with exercise load quantification for plantar health evaluation, this paper presents the prototype of an integrated bio-physiological measurement system based on the BSN for long-term plantar pressure measurement with SF and HR recording synchronously. In addition, a novel approach to exercise load quantification based on SF and HR is also proposed in this paper. System development and related methods are illustrated to provide an in-depth introduction to the challenges in this work. Preliminary experiments on 30 subjects were conducted to examine the capabilities of the measuring system.

The remaining of this paper is organized as follows. “Methods” Section presents the details of the prototype including hardware and software systems. The proposed method for plantar health evaluation is also involved in this section. Experiments and related results are given in “Experiments” Section to evaluate the performance of this work. “Discussion” Section and “Conclusion” Section makes a discussion and draws a conclusion, respectively.

## Methods

The innovative contribution of this work is providing an approach to the long-term monitoring and evaluation of plantar conditions during exercise based on the proposed wearable system and methods, which may have potential values in the early detection and diagnose of plantar symptoms. In this section, this work is described from four aspects including system overview, hardware system, software system and related methods for the evaluation of plantar conditions.

### System overview

**Fig. 1:**
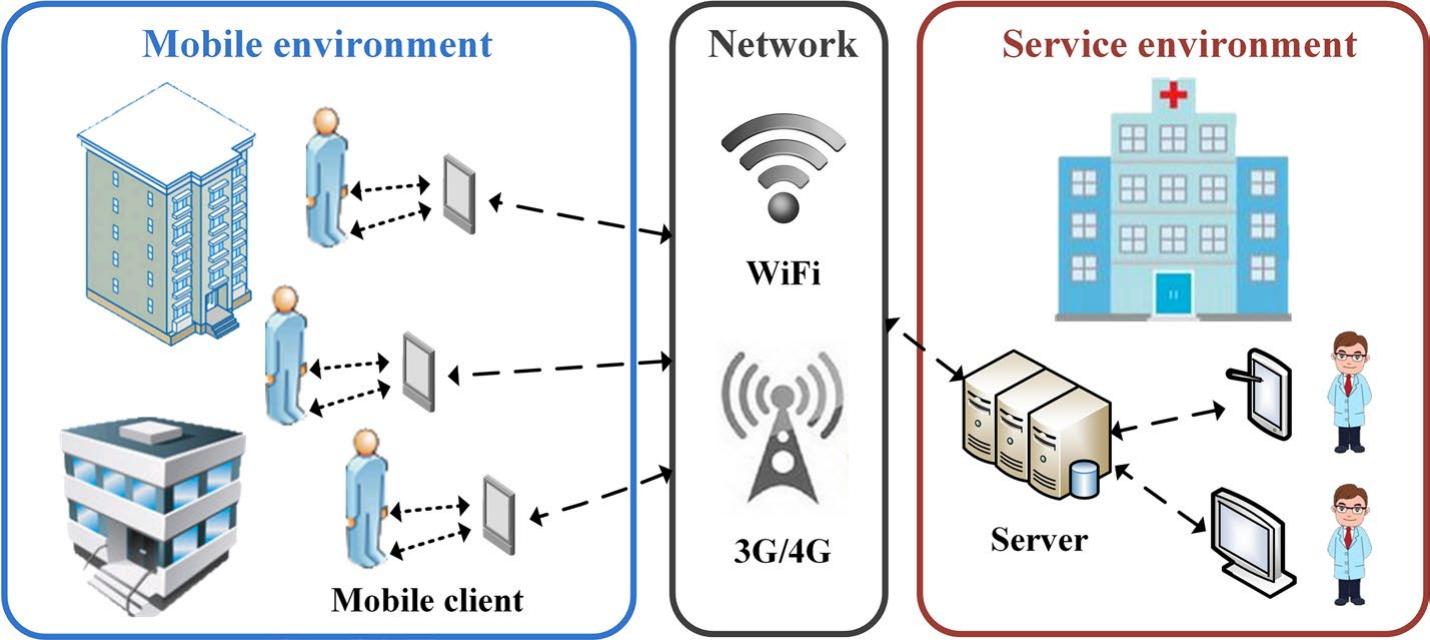
Framework of the proposed BSN-based system.

To guarantee the long-term monitoring without restrictions of environments, the proposed system is developed based on the framework of BSN as shown in Fig.  1. In the mobile environment, users are required to wear mobile clients for data acquisition and logging during activities. The corresponding information is transferred to the service environment via wireless networks such as wireless fidelity (WiFi) and 3rd/4th generation (3G/4G) telecommunications. In the service environment, servers are used for data storage and analysis. Experts can achieve related information of multiple users from server applications. The remaining of this section mainly described the proposed architecture and algorithms involved in the mobile client and BSN network.

### Hardware system

In Fig.  1, the main components of our proposed system are the mobile client, network and server, respectively.

### Mobile client

**Fig. 2:**
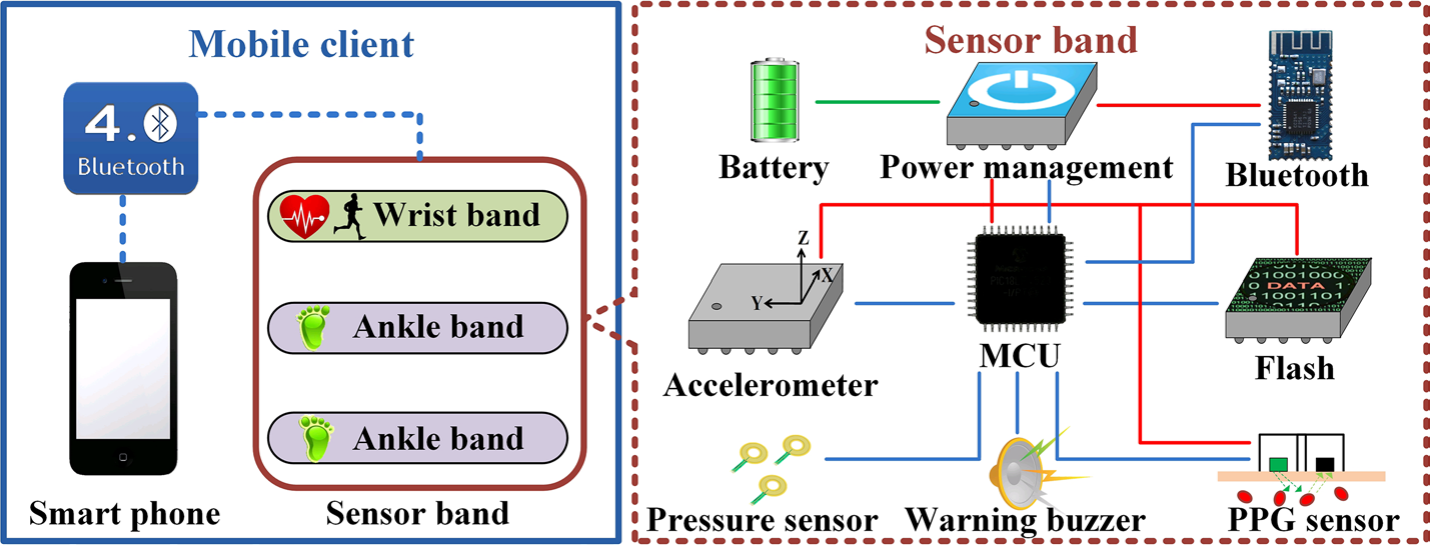
Architecture of the mobile client.

**Fig. 3:**
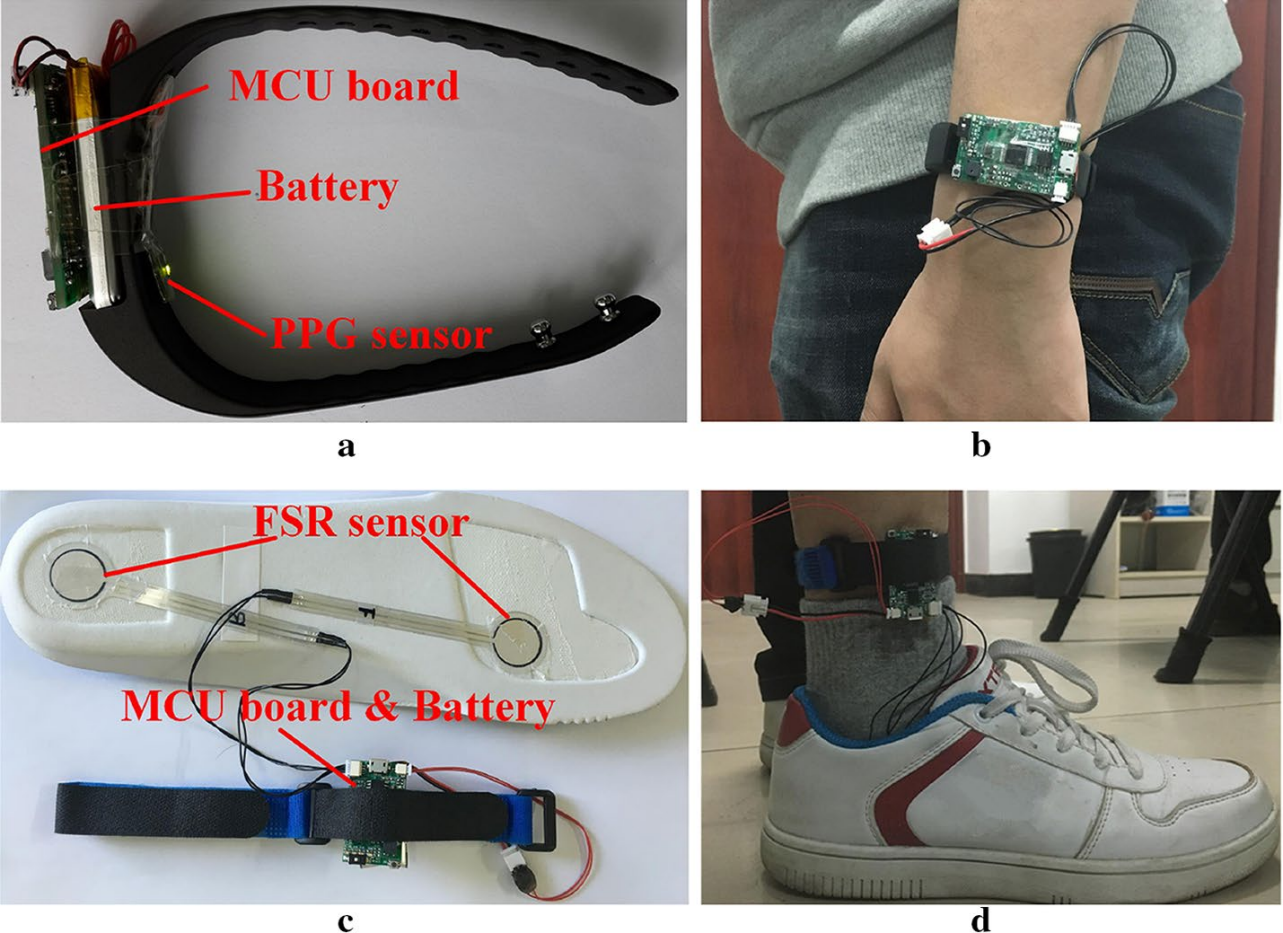
Sensor bands of the mobile client. **a** and **c** are the wrist-type band and ankle-type band, respectively. **b** and **d** give examples of wearing methods for (**a**) and (**c**), respectively.

The mobile client contains a smart phone and a series of sensor devices as illustrated in Fig.  2. Taking the convenience and comfortableness of wearing into account, all sensor devices using LiPoly batteries as the power supply are designed in the type of bands, including a wrist-type band for SF and HR recording, and two ankle-type bands for plantar pressure measurement. Communications between the smart phone and sensor bands are implemented via Bluetooth. An integrated micro controller unit (MCU) is used for vital feature extraction and system management. Considering the unnecessary energy consumption of real-time data transfer, we use flash-memory chips to store the acquired data on sensor bands firstly. As the monitoring is completed, the logged data is sent back to smart phones following designated sequential order. The prototypes and wearing methods of sensor bands are demonstrated in Fig.  3.

As shown in Fig.  3a, b, the wrist-type band with a PPG sensor and an accelerometer is developed for HR monitoring and SF estimation. A reflectance-mode LED/PD sensor is chosen as the media to acquire PPG signals. To obtain the signals with high quality, a LED with green light (560 nm) is selected as the light source of the sensor [28]. A tri-axial accelerometer is also integrated as a motion sensor in the wrist-type band.

As shown in Fig.  3c, d, the ankle-type band with pressure sensors distributed under insoles are employed for in-shoe plantar pressure recording. The literature [29] reveals that the heel and metatarsal areas of the foot bear higher pressure during activities. Intensified pressure changes in these two areas are likely indicators of plantar lesions such as neuropathy and ulceration. Hence, two patch force-sensitive resistors which have been calibrated are attached under the heel and first metatarsal area for pressure measurement in the proposed ankle-type band.

As the mobile terminal, a smart phone is mainly utilized to collect the recorded information and upload data to the server. The selected phones are compatible with protocols of Bluetooth 4.0, WiFi and 3G/4G to realize wireless communications with sensor bands and servers.

### Server and network

Compared with smart phones, servers are more capable for data analysis due to the enormous abilities of computation and storage. Mobile clients and servers are communicated through different kinds of networks based on different situations. The WiFi-based network is utilized for indoor exercise monitoring. In outdoor environments, 3G/4G networks are adopted as effective solutions. Networks are automatically switched with the priority of WiFi-based solution in our system.

### Software system

The system is designed to support acquisition, transmission, storage and analysis of related information from users. The developed software system is presented in terms of data measurement and user applications, respectively.

#### Data measurement

**Fig. 4:**
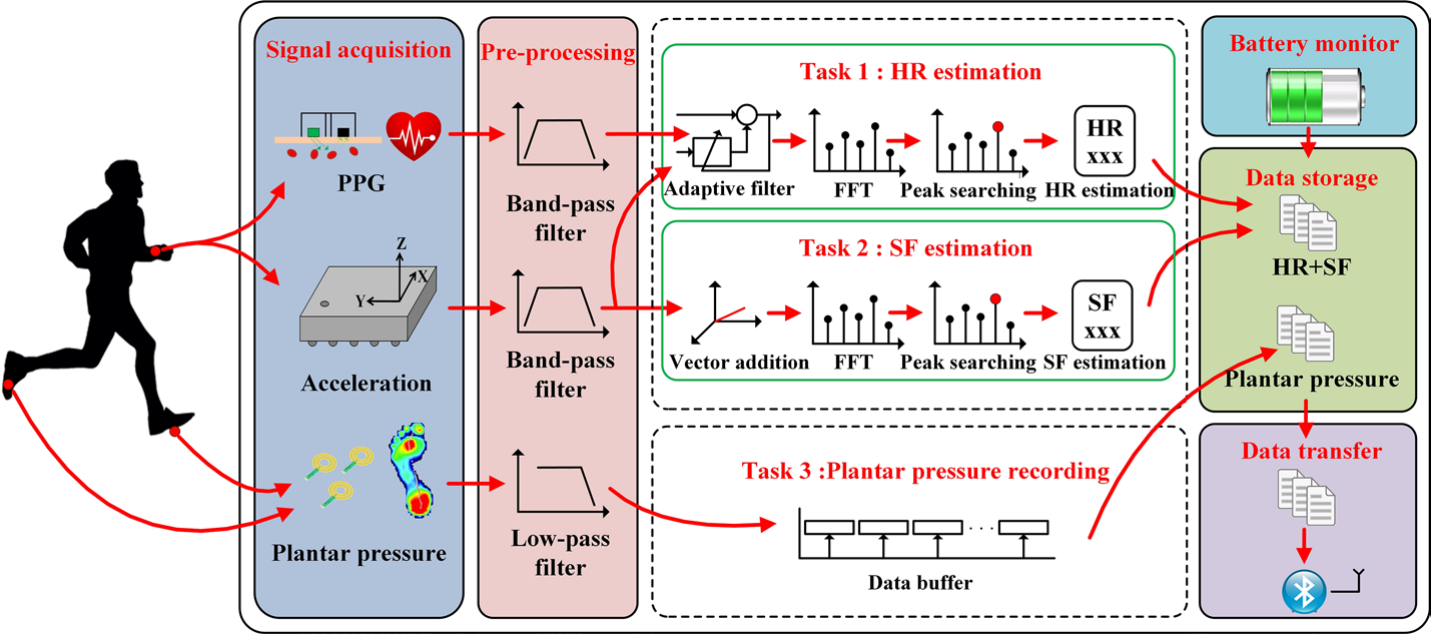
Software architecture of sensor bands.

As signal acquisition equipment, sensor bands contained in mobile clients play significant roles in the monitoring. For the sake of enhancing the system stability, the framework of programs is developed under the architecture of a real-time operating system (OS) named RTX OS to guarantee the effectiveness of multi-task scheduling [30]. The software architecture of sensor bands including three parallel bio-physiological measurements and other related functions is illustrated in Fig.  4.

**Fig. 5:**
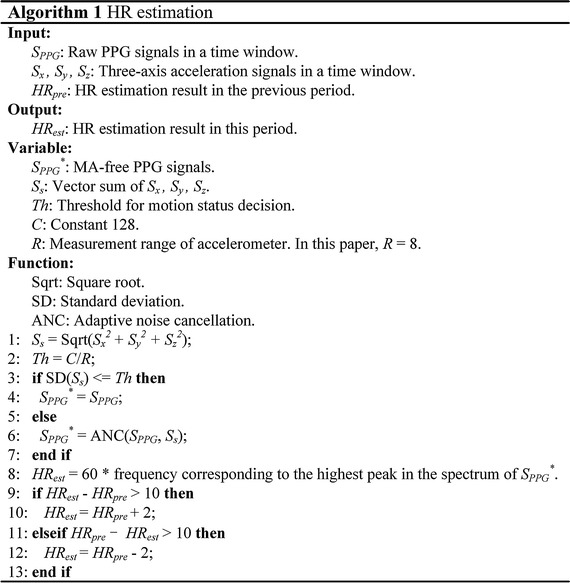
Algorithm of HR estimation.

Task 1 in Fig.  4 is HR estimation, which is important to the assessment of cardiovascular conditions. In our work, HR estimation is realized based on a single-channel PPG signal and the sum of tri-axial acceleration vector signals acquired from the wrist-type band. Algorithm 1 in Fig.  5 shows the major procedure of HR estimation. All signals are sampled at the rate of 100 Hz. In order to remove the components of high frequency noise and baseline drift, a band-pass filter with the cut-off frequencies of 0.2 and 10 Hz is employed, which is sufficient to cover the normal frequency range of HR [31]. A 3-s sliding time window is set on PPG and acceleration signals. The short window can make the proposed method keep track of the details of HR variabilities.

When the standard derivation (SD) of acceleration is lower than *Th* shown in Fig.  5, the filtered PPG signals are considered as MA-free signals. MA removal should be otherwise conducted primarily. *Th* is set as 16 based on the measurement range of accelerometers. Algorithms, such as TROIKA [32] and JOSS [33], have been proposed to suppress the MA interference and received remarkable progress in this area. Nevertheless, the required computational complexity of these approaches is the major resistive factor for implementation on current wearable devices with severely constrained capacity and speed of calculation, at least not in a real-time fashion. Alternatively, the low-complexity algorithm of adaptive noise cancellation [27] is adopted for MA removal. After achieving MA-free signals, the highest peak from the magnitude spectrum of PPG is selected as the dominant frequency related to HR estimates. Impulsive motion may disturb the signal track of the heart beat additively. To prevent acute fluctuations in the estimation between two nearby time windows, a regularization based on the comparison with previous temporal segment of estimate can be performed as illustrated in Fig.  5.

**Fig. 6:**
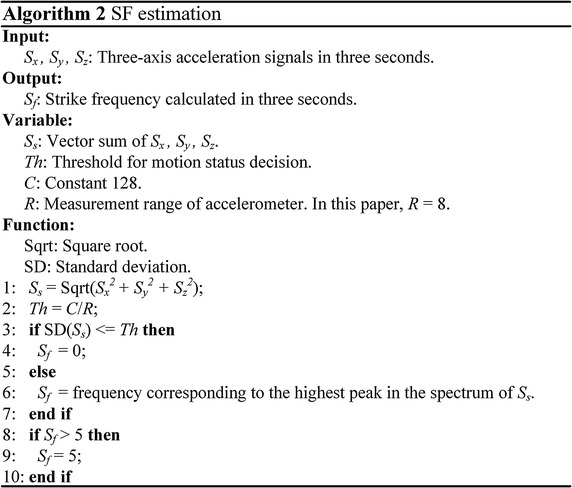
Algorithm of SF estimation.

Task 2 in Fig.  4 is the estimation of SF, which is a parallel measure to the intensity of activity. In consistent with the procedures of HR estimation, the sum of tri-axial acceleration vector signals is applied to identify effective steps. In a sliding window of 3 s, the spectrum and SD of the filtered acceleration signals are calculated. As shown in Fig.  6, the component related to the highest peak of the magnitude spectrum is considered as the dominant frequency if the SD is larger than *Th*. Since SF of an adult during activities is usually in the range of 0.5–5 Hz [34], the upper bound of SF is set to be 5 Hz in a time window.

Task 3 in Fig.  4 is plantar pressure recording, which is an important part of the foot health evaluation. As stated in SF estimation, the common SF is lower than 5 Hz. Hence, pressure sensors wired to the ankle-type band are synchronously sampled at 50 Hz which is sufficiently sensitive to subtle changes of the plantar pressure. A low-pass filter with the cut-off frequency of 10 Hz is utilized to remove the high-frequency noise before data storage.

Besides the stated functions, a battery monitor is also implemented for the warning of low power and the recording of energy consumption based on the data acquired from the power management chip.

#### User applications

**Fig. 7:**
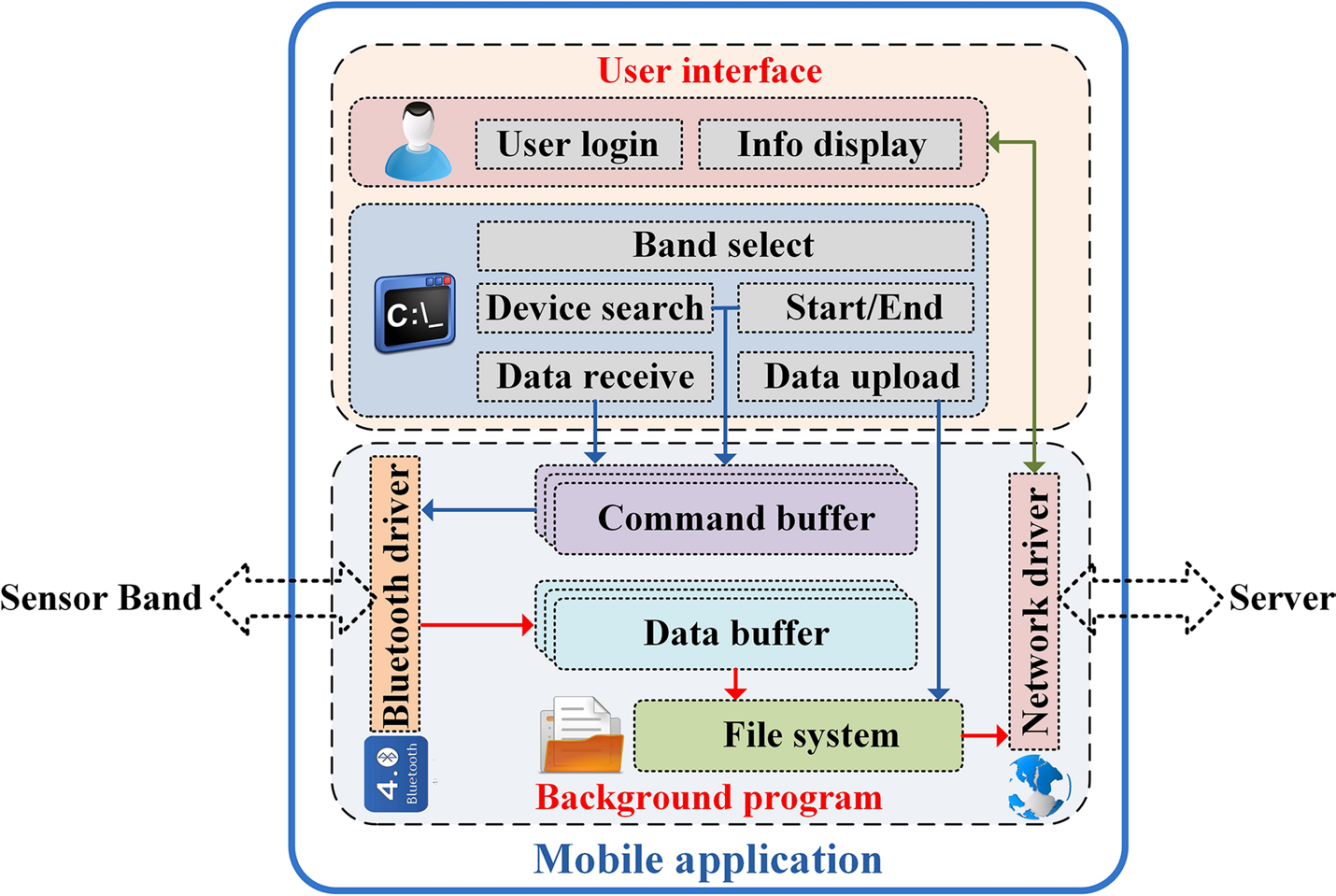
Architecture of the applications on smart phones.

Comprised of user interfaces and background programs, the developed application on smart phones is illustrated in Fig.  7. The corresponding contents involved in applications are described subsequently.

**Fig. 8:**
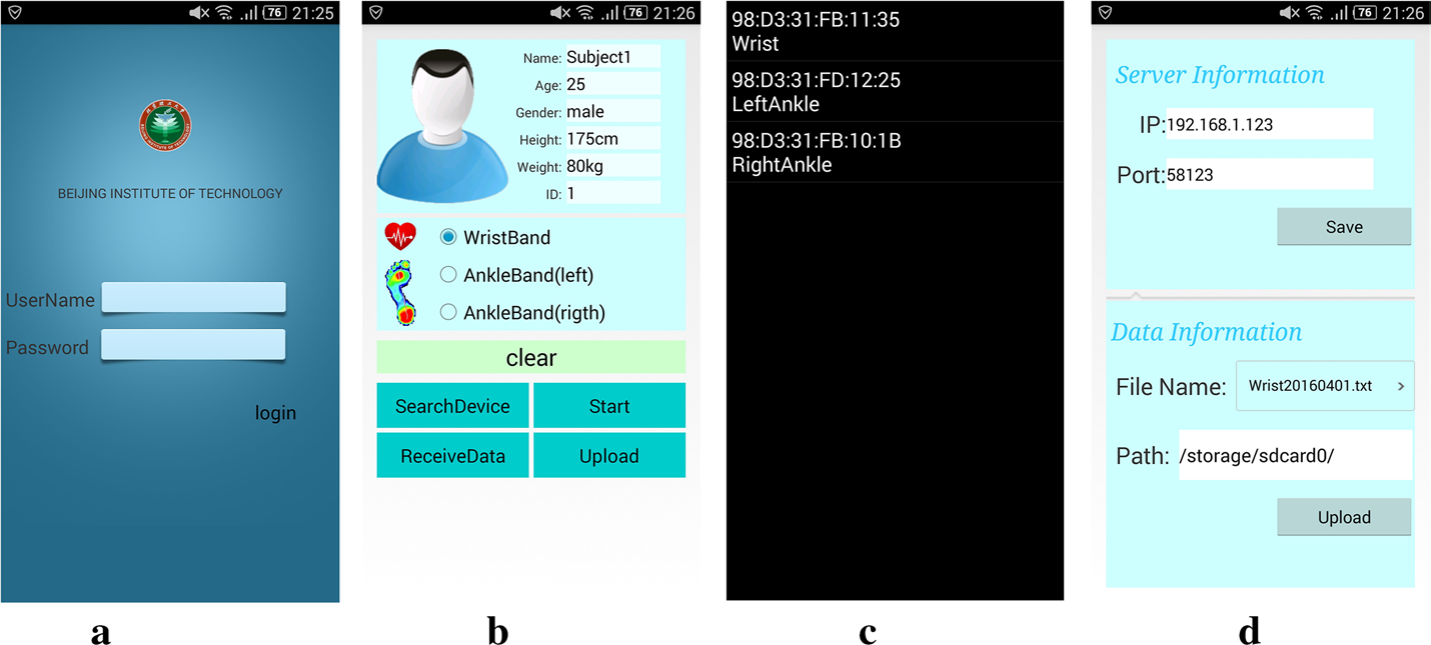
Software interfaces of mobile application on smart phones. **a** user login; **b** configuration; **c** list of searched devices; **d** upload information.

In our work, the mobile application as shown in Fig.  8 is developed based on the platform of Android 4.4. Aiming at improving the system security, only the users who get authentication from servers through the username and password can login the application as shown in Fig.  8a, b is the configuration interface where we can control the procedures of exercise monitoring. To start the monitoring, select one sensor band and search for the corresponding device name as listed in Fig.  8c. After the initial configuration, a start command is sent via the paired Bluetooth driver subsequently. The recorded information from sensor bands is stored in the file system of mobile applications, and it can be uploaded to the server manually off-line or automatically in real time. Basic information of servers and recorded data should be correctly configured before data uploading as demonstrated in Fig.  8d.

### Plantar condition evaluation

#### Data synchronization

**Fig. 9:**
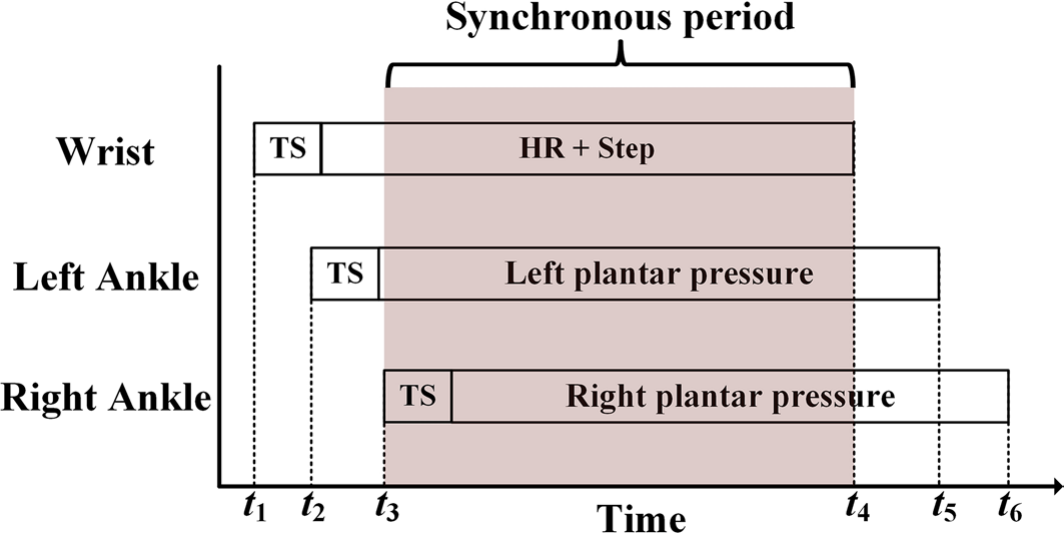
An example of synchronous period selection.

Due to asynchronous starting time of each sensor band in the BSN, temporal misalignment of the recorded vital time segments is considerable impact on the result of conjoint analysis. In order to overcome this drawback, a global timestamp (GTS) is included in the start command. The beginning of data stored in sensor band will use the GTS as a package header. Therefore, each piece of physiological recording can be synchronized by selecting the overlapped period as illustrated in Fig.  9, where* t*_1_–*t*_3_ and *t*_4_–*t*_6_ represent the beginning and ending of the monitoring on each band, respectively. The range of synchronous period, which is selected for the final conjoint analysis, is between *t*_3_ and* t*_4_ .

#### Exercise load quantification

The proposed approach to exercise load quantification is based on the combination of SF and HR. The ratio of SF ($R_{sf}$) defined in Eq.  (1) is adopted to describe the intensity of activity, where $S_f(i)$ and *L* represent the $i^{th}$ SF and the total number of time windows. $S_{fmax}$ is set as 5 since SF is usually in the range of 5 Hz  [29]. Any frequency values larger than $S_{fmax}$ are regarded as $S_{fmax}$ practically.

1 $$\begin{aligned} R_{sf}(i)=\frac{S_f(i)}{S_{fmax}},~1\le {i}\le {L} \end{aligned}$$

HR is employed to evaluate the exercise endurance of users. Denoted by $R_{hr}$, the ratio of HR is defined in Eq.  (2) where $H_r(i)$ represents the $i^{th}$ estimated HR. The value of $H_{rmax}$ and $H_{rmin}$ are configured as 180 and 50 beats per minute (BPM) to cover the normal range of HR [26]. Any HR value beyond the restrictive range are set as the upper bound practically. Since HR is a non-zero value which is different from SF, $H_{rmin}$ is adopted in the normalization to keep $R_{hr}$ in a wide dynamic range between 0 and 1.

2 $$\begin{aligned} R_{hr}(i)=\frac{H_r(i)-H_{rmin}}{H_{rmax}-H_{rmin}},~1\le {i}\le {L} \end{aligned}$$

With the predefined $R_{sf}$ and $R_{hr}$, exercise load is graded by *S* as defined in Eq.  (3). Taking Borg’s RPE [13] as the reference, we divided the amount of load into four levels from no load to intensive load based on the range of scores as shown in Table 1. Example activities are listed in the table to make a supplementary illustration of load intensities. Compared with the RPE, the proposed method is much more objective for exercise load quantification.

3 $$\begin{aligned} S(i)=100R_{sf}(i)R_{hr}(i),~1\le {i}\le {L} \end{aligned}$$

**Table 1:** Four levels of exercise load quantification.

Level	Score	Load description	Borg’s RPE	RPE description	Example
1	0	No load	–	Nothing at all	Sitting
2	1–20	Light load	6–10	Very, very light–very light	Slow walking
3	21–50	Moderate load	11–14	Fairly light–somewhat hard	Fast walking
4	51–100	Intensive load	15–20	Hard–very, very hard	Running

#### Analysis of plantar pressure

In order to make comparisons among all subjects, normalized plantar pressure is used in this paper to avoid the variations of absolute pressure caused by individual factors such as height and weight  [35]. Denoted by $P_n$, normalized plantar pressure is defined as Eq.  (4):

4 $$\begin{aligned} P_n(i,j)=\frac{P(i,j)}{P_{max}},~1\le {i}\le {4},~1\le {j}\le {K} \end{aligned}$$

where *P*(*i*, *j*) is the $j^{th}$ pressure value of the $i^{th}$ sensor, and $i=1,2,3,4$ represent the first metatarsal area of left foot (MoL), the heel of left foot (HoL), the first metatarsal area of right foot (MoR) and the heal of right foot (HoR), respectively. $P_{max}$ is the largest value recorded from all sensors. *K* is the number of recordings. $P_{nth}$ defined in Eq.  (5) is proposed to identify the noticeable difference of pressure values. In this paper, we set a criterion that potential plantar bruise may occur on the user’s foot if $P_{nth}$ is larger than 0.5. Combined with exercise load quantification, the magnitude of $P_{nth}$ can also be employed to reflect the severity of potential bruise. Drastic changes with large $P_{nth}$ during low-score exercise usually reveals worse conditions of plantar symptoms.

5 $$\begin{aligned} P_{nth}(k)=\max (P_n(i,k)-P_n(j,k)),~1\le {i,j}\le {4},~1\le {k}\le {K} \end{aligned}$$

To demonstrate the average difference of pressure dynamics among all sensors, normalized pressure distribution [29] denoted by $P_d$ is introduced into plantar evaluation as shown in Eq.  (6):

6 $$\begin{aligned} P_d(i)=\frac{P_a(i)}{P_{amax}},~1\le {i}\le {4} \end{aligned}$$

where $P_a(i)$ was temporally accumulated pressure recorded from the $i^{th}$ sensor in each status, and $P_{amax}$ is the maximum value of $P_a$. $P_{dth}$ defined in Eq.  (7) is similarly set to reflect the notability of difference and a threshold of 0.5 is also given as a criterion to identify the potential bruise.

7 $$\begin{aligned} P_{dth}(i)=\max (P_d(i)-P_d(j)),~1\le {i,j}\le {4} \end{aligned}$$

## Experiments

In this section, the proposed system was examined on 30 subjects to verify the capabilities of conjoint analysis between plantar pressure and exercise load quantification. All experiments on human had been reviewed by the ethical committee of China-Japan Friendship Hospital (No. 2013-8) and all subjects were informed with.

### Experimental setup

Thirty subjects (19 males and 11 females, $25.3\pm 2.1$ years old) were invited to the experiments including two subjects with bruise under HoL and MoL, respectively. Moreover, Subject 6 (S6) with bruise under HoL had more severe symptoms than Subject 15 (S15) with the bruise under MoL according to the expert’s evaluation. For the illustrative purpose of examining the measuring accuracy on the specific motional and physiological parameters, a commercial Holter ECG recorder Philips DigiTrak Plus 3100A and a wrist-type band of Fitbit flex for the analysis of SF were chosen to form a comparative sensory system that perform simultaneous data logging. The results from this combination were considered as the ground truth of the accuracy evaluation. To improve the reliability of evaluation results, the ground truth of HR was carefully examined by manually labeling and counting the R peaks over the entire recorded ECG traces. SF was only calculated every minute because the Fitbit flex can only record step counts in a fixed period. To simulate the daily activities, experiments were conducted in outdoor environments. Initial contents of test comprised 10 minutes of chair sitting, 10 minutes of walking and 10 minutes of jogging to access the measurement performance in static and simple motional actions. The recorded data were read back and uploaded to the server through smart phones.

### Results

The summary of experimental results including measuring accuracy, scores of exercise load, differences of plantar pressure distribution and related information about subjects were presented in Table 2. All related details are described in the subsequent contents.

**Table 2:** Summary of experimental results from 30 subjects.

S	G	A	mRaeHR	mRaeSF	mSW	mSJ	mPnthW	mPnthJ	PdthW	PdthJ
1	M	27	3.72	3.49	10.45	49.71	0.44	0.13	0.48	0.15
2	M	25	4.12	6.77	14.18	59.65	0.23	0.11	0.22	0.17
3	F	25	4.79	4.88	13.37	71.54	0.25	0.09	0.21	0.12
4	M	22	4.41	3.27	35.60	81.85	0.26	0.14	0.25	0.15
5	F	27	3.95	4.70	17.06	68.70	0.30	0.13	0.27	0.10
6	M	26	4.54	4.57	17.03	69.34	*0.62*	*0.68*	*0.53*	*0.73*
7	M	23	4.42	5.54	19.37	72.37	0.28	0.06	0.23	0.04
8	F	22	3.38	6.10	13.46	71.20	0.36	0.24	0.33	0.16
9	F	23	2.75	6.28	18.35	72.16	0.09	0.08	0.11	0.13
10	M	24	4.01	7.44	31.71	75.12	0.25	0.12	0.27	0.11
11	M	27	5.62	3.39	16.93	58.97	0.23	0.05	0.19	0.08
12	M	28	4.39	4.71	19.06	73.55	0.18	0.11	0.26	0.13
13	F	26	5.50	5.67	10.03	50.50	0.14	0.05	0.24	0.05
14	M	25	3.58	5.92	40.05	76.57	0.18	0.09	0.09	0.02
15	M	32	3.01	2.16	28.30	66.53	0.48	*0.66*	0.46	*0.64*
16	F	25	6.57	2.15	15.81	74.83	0.15	0.20	0.11	0.17
17	M	25	6.49	4.06	32.23	77.60	0.14	0.06	0.08	0.08
18	F	23	5.16	2.11	12.19	71.96	0.07	0.12	0.20	0.11
19	F	24	3.32	5.00	9.51	71.30	0.22	0.07	0.21	0.06
20	M	25	4.50	4.42	15.67	68.74	0.30	0.14	0.30	0.10
21	M	24	4.70	1.70	18.42	59.54	0.12	0.09	0.09	0.14
22	M	24	10.17	4.37	44.53	82.49	0.35	0.14	0.33	0.08
23	F	26	3.98	5.68	11.86	74.73	0.08	0.13	0.09	0.12
24	M	29	5.69	7.09	34.98	76.08	0.22	0.05	0.22	0.06
25	M	24	4.34	5.34	24.21	72.80	0.10	0.14	0.13	0.16
26	M	26	7.03	4.30	30.24	72.61	0.10	0.15	0.05	0.12
27	F	26	4.98	6.74	16.24	65.90	0.33	0.13	0.33	0.11
28	F	23	6.78	6.28	17.14	74.38	0.24	0.05	0.12	0.09
29	M	27	3.64	4.61	18.07	66.35	0.06	0.05	0.08	0.02
30	M	25	3.69	4.23	27.26	79.75	0.21	0.12	0.16	0.10

Values of mRaeHR and mRaeSF are expressed as percentages

Italic values indicate the significance of noticeable difference related to $P_{nth}$ and $P_{dth}$

*S* subject, *G* gender, *A* age, *M* Male, *F* female, *mRaeHR* mean $R_{AE}$ of HR, *mRaeSF* mean $R_{AE}$ of SF, *mSW* mean score of exercise load in walking status, *mSJ* mean score of exercise load in jogging status, *mPnthW* mean value of $P_{nth}$ in walking status, *mPnthJ* mean value of $P_{nth}$ in jogging status, *PdthW* value of $P_{dth}$ in walking status *PdthJ* value of $P_{dth}$ in jogging status

Measuring accuracy of HR and SF were examined in terms of the correlation and the ratio of absolute error to make exercise load quantification reliable. Denoted by *r* in Eq.  (8), the coefficient of correlation measured between the parametric estimate *E* produced by the system and the ground truth *T* is defined to be:

8 $$\begin{aligned} r=\left| \frac{\sum \limits _{i=1}^N(E(i)-\bar{E})(T(i)-\bar{T})}{[\sum \limits _{i=1}^N(E(i)-\bar{E})^2\sum \limits _{i=1}^N(T(i)-\bar{T})^2]^{\frac{1}{2}}}\right| ,~1\le {i}\le {N} \end{aligned}$$

where *E*(*i*) and *T*(*i*) represent the $i^{th}$ *E* and *T*. $\bar{E}$, $\bar{T}$ and *N* are the average of *E* and *T*, and the number of data, respectively. The more correlated between *E* and *T*, the large *r* would be.

The ratio of absolute error ($R_{AE}$) defined in Eq.  (9) was also given to evaluate the performance of measurements. $R_{AE}$ would be disproportional to the change of measurement accuracy and stability.

9 $$\begin{aligned} R_{AE}(i)=\left| \frac{E(i)-T(i)}{T(i)}\right| ,~1\le {i}\le {N} \end{aligned}$$

**Fig. 10:**
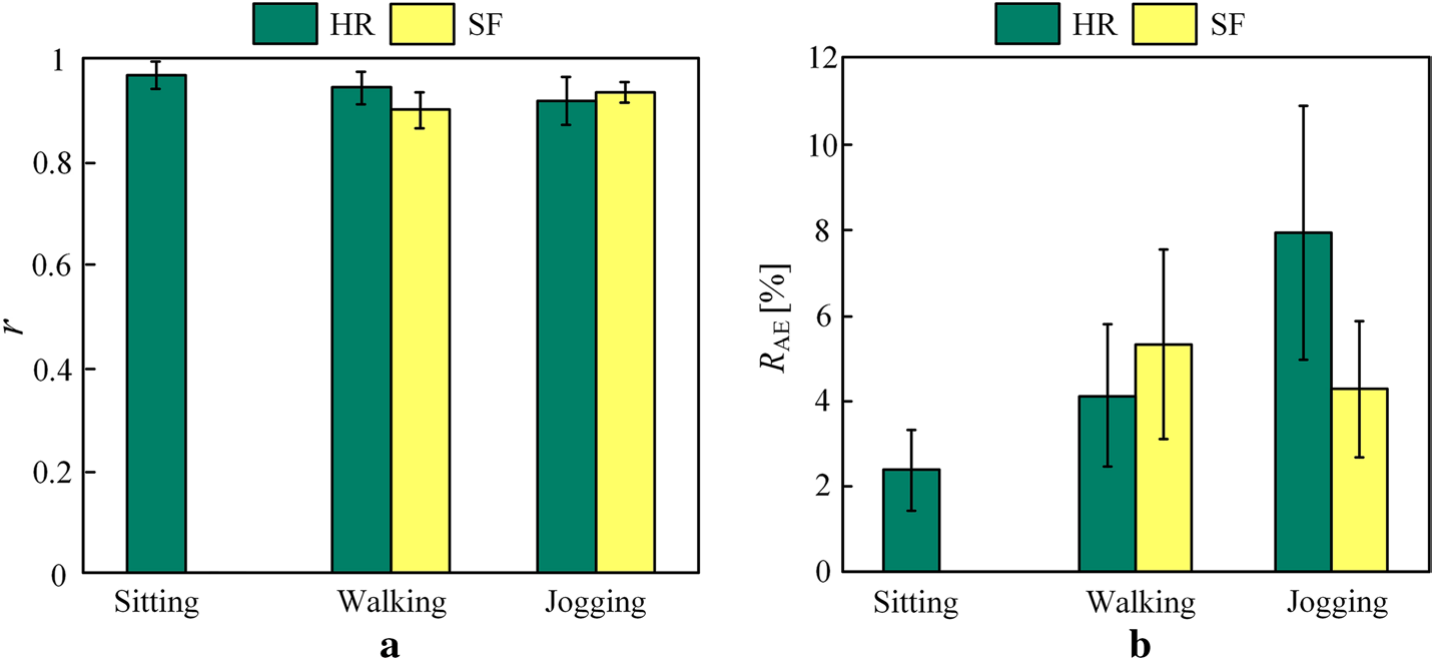
*r* and $R_{AE}$ of HR and SF estimation on all subjects’ recordings.** a** *r*;** b** $R_{AE}$.

In the first set of evaluation, the *r* between the estimates and ground truth is illustrated in Fig.  10a. The corresponding *r* values regarding HR were $0.97\pm 0.03$, $0.94\pm 0.03$ and $0.92\pm 0.05$ during each motion status. Since the recorded numbers of SF were unaccounted during chair sitting, we ignored the analysis in this status. The *r* values regarding SF were $0.90\pm 0.04$ and $0.93\pm 0.02$ during walking and jogging status, respectively. Values of $R_{AE}$ calculated from each status were given in the Fig.  10b. The $R_{AE}$ regarding HR were $0.02\pm 0.01$, $0.04\pm 0.02$ and $0.08\pm 0.03$ during each status. And $R_{AE}$ of SF were $0.05\pm 0.02$ and $0.04\pm 0.02$ during walking and jogging status, respectively. It was found that impact induced from the MA on HR estimation became increasingly noticeable. On the contrary, more precise results were achieved in SF estimation due to the higher signal-noise ratio of acceleration signals as the motions were intensified. Generally, the high correlation and low error rate revealed that the prototype system was effective on HR and SF estimation, laying a reliable foundation for exercise load quantification.

**Fig. 11:**
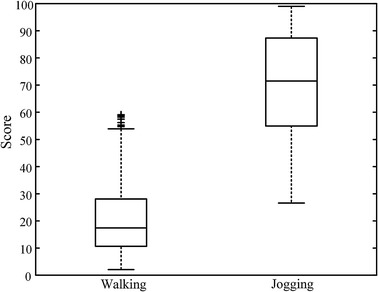
Box plot of scores in different motion status.

The score of exercise load was obtained on all subjects’ recordings according to Eq.  (3). We calculated the average score every minute to suppress the interference of abnormal data. The statistical results including mean value, range and distribution in the predefined load levels were presented in Table 3. Considering that the values of SF were always zero during sitting, we ignored the analysis in this status. About 96.4% of score values in walking status were within the range of Level 2 and 3, revealing that walking was usually an activity with light or moderate load. There were 80.3% of scores in jogging status were in the range of Level 4, indicating that jogging was an intensive-load activity for most subjects. Figure  11 presents the box plot of scores in two different motion status. It is obvious that most of the scores were comprised in the range of 1–50 during walking status. And the most in jogging status were in the range of 51–100 though the dynamic range was larger than walking status. All stated results indicated the rationality of predefined level setting. It also revealed that the similar exercise may have different intensities for different subjects.

**Table 3:** Mean, range and distribution of scores in motion status.

Status	Mean	Range	L1 (%)	L2 (%)	L3 (%)	L4 (%)
Walking	21.1	2.1–59.1	0	60.7	35.7	3.6
Running	70.2	26.6–99.1	0	0	19.7	80.3

**Fig. 12:**
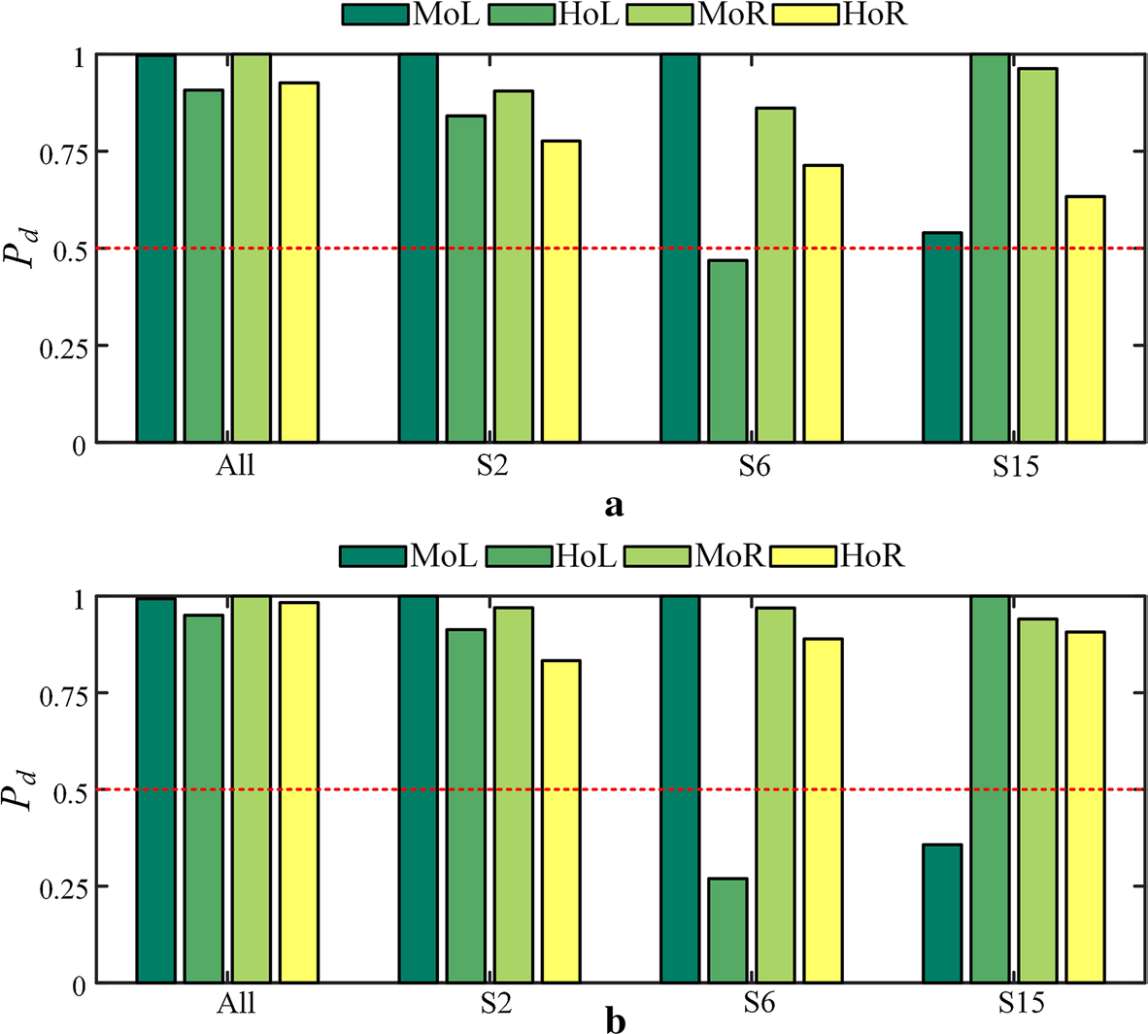
Distribution of plantar pressure in different motion status. **a** walking status; **b** jogging status.

Two groups of histograms reflecting the plantar pressure distribution in two different motion status were illustrated in Fig.  12. We compared the distribution ($P_d$) defined in Eq. (6) on all subjects’ recordings with $P_d$ of three representative subjects in Table 2 including a normal subject (S2) and two subjects with bruise under HoL (S6) and MoL (S15), respectively. It was found that the distribution of subjects with bruise were much more unbalance than others. Noticeable differences ($P_{dth}>0.5$) were occurred in S6 and S15 during jogging status as illustrated in Fig.  12b, indicating that there were potential bruises happened on both subjects. Compared with S15, S6 was supposed to have more severe symptoms since the noticeable difference was just happened on S6 during walking status which can be observed from Fig.  12a.

**Fig. 13:**
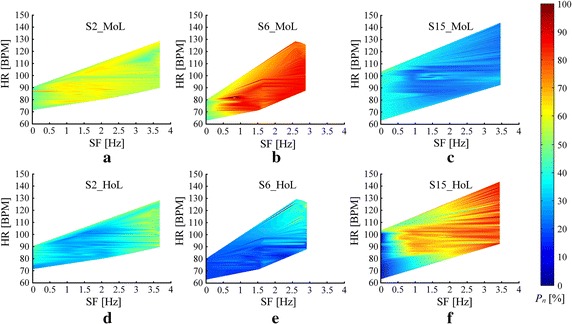
Comparison of PPV on the left foot among S2, S6 and S15. **a** and **b** illustrate the PPV of S2, **b** and **e**, **c** and **f** are the results of S6 and S15, respectively.

To identify the associated factors causing plantar pressure variability (PPV), a conjoint analysis was conducted using sampled plantar pressure, HR and SF. Figure  13 demonstrates the PPV from left foot of S2, S6 and S15. It can be observed that along with the enhanced motion intensity, increased HR and unbalanced change of plantar pressure from S6 and S15 occurred. It was also evidential that the foot area without bruise bore much more pressure during long-term activities.

**Fig. 14:**
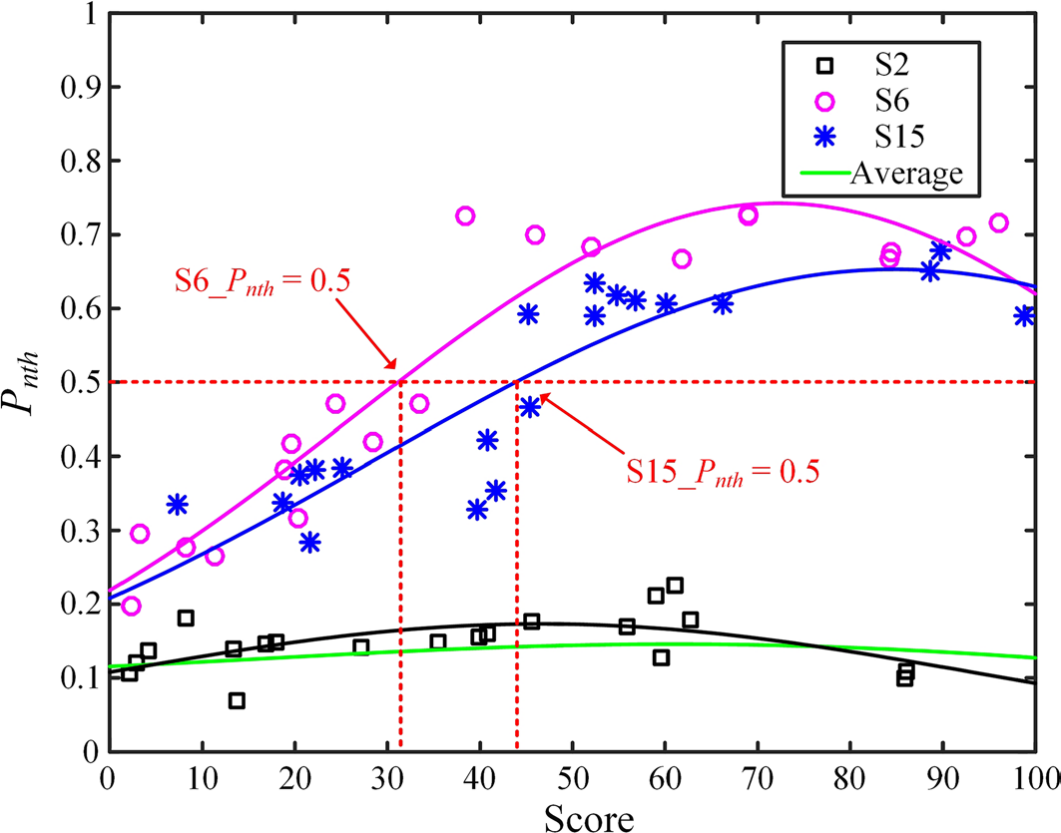
Comparison of $P_{nth}$ with different load exercise.

Combined with exercise load quantification, $P_{nth}$ defined in Eq.  (4) is an effective indicator to represent PPV in different motion status. Figure  14 presents the trend of pressure difference among S2, S6 and S15. Average $P_{nth}$ of all subjects was also given as the reference for comparison. With Gauss equation adopted, fitted curves were calculated based on the discrete data of each subject. Compared with the predefined criterion, drastic changes ($P_{nth}>0.5$) of pressure happened on S6 and S15. The scores of interpolations calculated from the fitted curves and the criterion were 31.1 and 43.8 for S6 and S15, indicating that S6 had more severe bruise because the drastic change occurred with lighter exercise. The fitted curve of S2 was close to the average trend revealing the normal PPV during exercise. These results agreed well with the analysis of plantar pressure distribution.

In addition to the capabilities of measurement, the system performance such as energy and time consumption were also analyzed in the experimental results. Table 4 tabulates the mean value with SD of energy and time consumptions on each band based on all subjects’ recordings. It can be observed that wrist-type bands consumed more energy than ankle-type bands due to the integrated PPG sensors. However, wrist-type bands consumed much shorter time to upload the recorded data from sensor bands to servers since it just stored the results of HR and SF, which was different from the raw pressure values logged on the ankle-type band.

**Table 4:** Consumptions of energy and data uploading time calculated from sensor bands.

Band type	Energy (mAh)	Time (s)
Wrist	83.4 ± 3.5	6.3 ± 0.2
Left ankle	42.1 ± 1.4	45.2 ± 0.2
Right ankle	42.7 ± 0.8	45.4 ± 0.1

All data are presented as mean±SD

## Discussion

The experiments conducted in this paper were raw but comprehensive performance measures of a prototype integrated bio-physiological system, suggesting both high system integrity and potentials to improve the development of plantar pressure evaluation with exercise load quantification. The estimated correlation rate with reference devices ($r>0.9$) and error rate on the average ($R_{AE}<0.08$) of HR and SF indicated equal measuring capability as the existing commercial products and an acceptable level of error rate. Comprised of the conjoint analysis with HR and SF, the proposed method for exercise load quantification was examined on all subjects’ recordings. The results revealed the effectiveness of quantification and the rationality of load level setting. Furthermore, the implication of foot bruise symptom reflected by the unusual pressure difference and distribution of pressure dynamics approved that the sensitivity of the proposed system was adequate to distinguish regional anomaly from healthy pressure response. At the same time, the combination of plantar pressure monitoring and exercise load quantification offered an approach to quantify the severity of bruise symptom during exercise.

However, there were still three limitations in this paper. Firstly, energy consumption of the wrist-type band was higher than expected, which had impact on the duration of battery life. Low energy techniques will be adopted to reduce energy consumption further. Secondly, more pressure sensors need to be integrated in the ankle-type band to make more precise analysis of plantar pressure especially the identification of potential bruise positions. Ultimately, due to the stage of prototype system, the experiments were just conducted on 30 subjects including 2 subjects with plantar bruise symptoms. More examinations on various subjects are necessary to further prove the effectiveness of the proposed system and methods.

## Conclusion

In this paper, the prototype of an innovative BSN-based bio-physiological measuring system has been implemented for the long-term monitoring and evaluation of plantar condition during exercise. Details of the prototype are illustrated in terms of hardware and software to prove the feasibility of the system. An approach to exercise load quantification based on HR and SF calculated from the sensor band has been proposed to assist in the analysis of plantar conditions. From the aspects of measuring precision and effectiveness on abnormal detection and assessment, we have conducted experiments on 30 subjects to preliminarily evaluate the capabilities of the prototype system. The experimental results indicated that the proposed system has a great potential value in the applications of plantar health evaluation.

## Abbreviations

BSN: body sensor network
SF: strike frequency
HR: heart rate
GPS: global positioning system
RPE: rating of perceived exertion
BP: blood pressure
PTT: pulse transit time
MA: motion artifact
ECG: electrocardiography
PPG: Photoplethysmography
LED: light-emitting diode
PD: photo diode
WiFi: wireless fidelity
3G/4G: 3rd/4th generation
MCU: micro controller unit
OS: operating system
SD: standard derivation
GTS: global timestamp
BPM: beats per minute
MoL: first metatarsal area of left foot
HoL: heel of left foot
MoR: first metatarsal area of right foot
HoR: heel of right foot
